# Supplementary material for: Acute hypothalamic suppression significantly affects trabecular bone but not cortical bone following recovery and ovariectomy surgery in a rat model
Source: PeerJ. 2016 Jan 12;4:e1575. doi: 10.7717/peerj.1575 (PMC4715452; doi:10.7717/peerj.1575)
Supplement: Supplemental Information 1 [file peerj-04-1575-s001.pdf]

Animal		Day of VO	BW@VO	BW @SAC	Uterus	Ovaries	Retro Fats	Gonadal Fat Pads	Total Fat
1	C-FR1	29	82.2	184	0.2583	0.1305	1.6232	1.1884	2.8116
2	C-FR2	34	117.45	195.9	0.3309	0.1425	1.9532	1.3151	3.2683
3	C-FR3	31	91.25	196.5	0.3769	0.1266	2.287	1.1802	3.4672
4	C-FR4	34	114.6	202.3	0.2753	0.1011	2.1198	1.6168	3.7366
5	C-FR5	32	89.5	181.1	0.4362	0.096	3.28	2.2787	5.5587
6	C-FR6	36	103.2	180.1	0.2366	0.0858	2.0492	2.018	4.0672
7	C-FR7	31	95.78	209.9	0.4129	0.1685	2.7723	2.3752	5.1475
8	C-FR8	32	92.8	175.2	0.4966	0.129	1.0184	0.8024	1.8208
9	C	37	114.4	161.2	0.2521	0.0795	0.3203	0.4844	0.8047
10	C	36	136.6	201.3	0.4967	0.1398	0.7412	0.9574	1.6986
11	C	39	117.2	155.8	0.2908	0.082	0.2195	0.3924	0.6119
12	C	37	116.9	175.8	0.2533	0.0586	0.4775	0.7023	1.1798
13	C	34	116.2	189.3	0.4366	0.1057	1.1309	1.1522	2.2831
14	C	33	92.6	163.9	0.6566	0.0972	0.3769	0.4792	0.8561
	**AVG**	**33.92857**	**105.7629**	**183.7357**	**0.372129**	**0.1102**	**1.454957**	**1.210193**	**2.66515**
	**SD**	**2.81382**	**15.39687**	**16.44888**	**0.123856**	**0.030156**	**0.983191**	**0.653431**	**1.608298**
	**CV**	**8.293364**	**14.55792**	**8.952466**	**33.28311**	**27.365**	**67.57525**	**53.99397**	**60.34551**
45	FR-G	36	108.1	164.8	0.0402	0.0242	0.4804	0.5021	0.9825
46	FR-G	50	159.1	159.1	0.0279	0.0227	0.7918	0.9893	1.7811
47	FR-G	50	155.2	155.2	0.1153	0.044	0.3734	0.2611	0.6345
48	FR-G	50	165.4	165.4	0.0213	0.0314	0.5608	0.6408	1.2016
49	FR-G	36	99.2	153.4	0.0445	0.0231	0.9638	0.9908	1.9546
50	FR-G	50	170.7	170.7	0.0515	0.0333	0.3199	0.4413	0.7612
51	FR-G	41	125.3	155.1	0.157	0.0356	0.7046	0.8786	1.5832
52	FR-G	50	169.2	169.2	0.089	0.0348	0.1123	0.0763	0.1886
	**AVG**	**45.375**	**144.025**	**161.6125**	**0.068338**	**0.031138**	**0.538375**	**0.597538**	**1.135913**
	**SD**	**6.566963**	**28.79701**	**6.78179**	**0.047786**	**0.00744**	**0.27599**	**0.339621**	**0.609878**
	**CV**	**14.47265**	**19.99445**	**4.196328**	**69.9267**	**23.89289**	**51.26353**	**56.83681**	**53.69057**
53	GNRH	50	225.6	225.6	0.0558	0.0429	1.5011	1.5033	3.0044
54	GNRH	49	209.3	209.3	0.0413	0.0414	1.1827	1.2411	2.4238
55	GNRH	38	141.4	206.7	0.0968	0.0706	0.8329	0.7208	1.5537
56	GNRH	50	217	217	0.05	0.0392	1.4862	1.2443	2.7305
57	GNRH	50	222.7	222.7	0.0952	0.0452	1.1507	1.2545	2.4052
58	GNRH	50	213.9	213.9	0.0295	0.0507	1.2647	1.5323	2.797
59	GNRH	50	202.8	202.8	0.1373	0.0563	0.8781	1.1992	2.0773
60	GNRH	37	125.4	183.4	0.2588	0.0518	1.1235	0.7772	1.9007
61	GNRH	40	165.6	208.6	0.3444	0.0968	1.4617	1.0207	2.4824
62	GNRH	50	216.8	216.8	0.0705	0.0235	1.0333	1.0817	2.115
63	GNRH	50	195.6	195.6	0.0652	0.0222	0.583	0.5015	1.0845
64	GNRH	37	124.1	190.9	0.1108	0.0414	0.4238	0.4023	0.8261
65	GNRH	50	183.2	183.2	0.0751	0.0156	0.6172	0.4432	1.0604
66	GNRH	50	224.5	224.5	0.0366	0.0228	0.7636	1.1278	1.8914

	**AVG**	**46.5**	**190.5643**	**207.2143**	**0.104807**	**0.044314**	**1.021607**	**1.003564**	**2.025171**
	**SD**	**5.626175**	**36.82402**	**14.35768**	**0.090195**	**0.021379**	**0.349476**	**0.374735**	**0.684125**
	**CV**	**12.0993**	**19.32367**	**6.928903**	**86.05812**	**48.24378**	**34.20848**	**37.3404**	**33.78111**
**15**	**C-R**	34	97.2	295.5	0.5807	0.0941	3.0818	5.1127	8.1945
**16**	**C-R**	35	110.1	338.4	0.5638	0.0964	3.3853	7.3772	10.7625
**17**	**C-R**	33	117.8	372.4	0.7499	0.1389	4.7111	6.9634	11.6745
**18**	**C-R**	35	117.8	305.2	0.6548	0.0963	3.0115	5.6044	8.6159
**19**	**C-R**	32	90.1	278.9	0.5193	0.0999	2.6292	4.6813	7.3105
**20**	**C-R**	38	138.8	296.7	0.6776	0.2213	1.9496	2.0276	3.9772
**21**	**C-R**	32	93.4	256.6	0.9028	0.0938	2.4529	2.7544	5.2073
**22**	**C-R**	34	106.6	324.7	0.4894	0.1207	5.9894	5.4591	11.4485
**23**	**C-R**	36	119.6	336.9	1.0334	0.3401	5.4391	6.8416	12.2807
**24**	**C-R**	33	103.8	349.5	0.5723	0.2209	6.3288	6.285	12.6138
**25**	**C-R**	33	99.9	259.9	0.4463	0.1659	2.092	2.6096	4.7016
**26**	**C-R**	36	110.7	315.9	0.5233	0.1447	3.1276	3.8948	7.0224
**27**	**C-R**	34	122.3	362.3	0.5641	0.0819	3.7939	16.6088	20.4027
**28**	**C-R**	33	101.8	300.1	0.6163	0.1318	5.534	6.907	12.441
**29**	**C-R**	36	125.2	334.4	0.7954	0.2509	6.847	6.7465	13.5935
	**AVG**	**34.26667**	**110.34**	**315.16**	**0.64596**	**0.153173**	**4.02488**	**5.99156**	**10.01644**
	**SD**	**1.709915**	**13.30692**	**34.85**	**0.162518**	**0.074191**	**1.637198**	**3.409605**	**4.252672**
	**CV**	**4.990025**	**12.05992**	**11.05788**	**25.15919**	**48.43578**	**40.67693**	**56.9068**	**42.45692**
**67**	**G-R**	58	207.3	324.1	0.6494	0.1508	6.9352	7.3922	14.3274
**68**	**G-R**	58	222.6	286.7	0.5431	0.1427	3.4925	5.6507	9.1432
**69**	**G-R**	54	226.9	350.4	0.455	0.191	6.0851	8.5871	14.6722
**70**	**G-R**	33	102.7	370.7	0.5396	0.1464	6.0241	8.6849	14.709
**71**	**G-R**	55	255.3	343.3	1.0954	0.2641	4.0277	5.1484	9.1761
**72**	**G-R**	43	203.7	371.9	0.6733	0.2838	6.4604	7.2009	13.6613
**73**	**G-R**	40	176.5	350.6	0.9822	0.203	4.9191	6.2808	11.1999
**74**	**G-R**	43	168.8	340.7	0.8708	0.0858	6.9868	4.3214	11.3082
**75**	**G-R**	43	203.7	325.2	0.8206	0.1577	4.6922	6.5867	11.2789
**76**	**G-R**	38	139.7	299.4	0.7588	0.0892	4.0474	3.906	7.9534
**77**	**G-R**	38	134.9	348.8	0.9245	0.1274	6.6443	4.5819	11.2262
**78**	**G-R**	48	241.53	372.5	0.9133	0.2531	6.3963	9.4726	15.8689
**79**	**G-R**	54	227.4	344.6	0.7007	0.2188	5.4293	7.1453	12.5746
**80**	**G-R**	38	142.1	391.3	0.6304	0.2212	8.9036	7.8948	16.7984
	**AVG**	**45.92857**	**189.5093**	**344.3**	**0.754079**	**0.181071**	**5.788857**	**6.632407**	**12.42126**
	**SD**	**8.434948**	**46.01618**	**28.66354**	**0.187411**	**0.062501**	**1.458177**	**1.734886**	**2.678159**
	**CV**	**18.36536**	**24.28175**	**8.325164**	**24.85292**	**34.51732**	**25.18938**	**26.15771**	**21.56108**
**30**	**C-OVX**	34	110.6	378.6	0.308		7.6513	6.4042	14.0555
**31**	**C-OVX**	32	107.9	344.5	0.3977		6.8079	3.8312	10.6391
**32**	**C-OVX**	33	97.9	335.8	0.4141		5.2888	3.9354	9.2242
**33**	**C-OVX**	34	121	422.2	0.2208		8.5861	8.0249	16.611
**34**	**C-OVX**	34	109.2	384.5	0.3322		6.0546	4.8302	10.8848
**35**	**C-OVX**	32	96.8	338.2	0.2753		5.5262	4.6057	10.1319

**36**	**C-OVX**	33	106.5	389.2	0.2847		10.6453	8.7322	19.3775
**37**	**C-OVX**	35	114.1	434.4	0.3231		10.3663	6.992	17.3583
**38**	**C-OVX**	34	123.6	407	0.1375		8.3669	9.05	17.4169
**39**	**C-OVX**	34	101.3	385.1	0.1871		5.6467	8.3354	13.9821
**40**	**C-OVX**	35	126.2	404.3	0.2704		3.6878	6.2976	9.9854
**41**	**C-OVX**	34	107.6	351.6	0.1561		3.7422	4.6097	8.3519
**42**	**C-OVX**	32	101.9	416.5	0.2959		4.7227	4.9469	9.6696
**43**	**C-OVX**	30	89.3	360.9	0.3441		5.7947	4.7729	10.5676
**44**	**C-OVX**	35	114.3	354.3	0.1548		5.7936	4.7861	10.5797
	**AVG**	**33.4**	**108.5467**	**380.4733**	**0.273453**	**#DIV/0!**	**6.57874**	**6.010293**	**12.58903**
	**SD**	**1.404076**	**10.30214**	**31.97592**	**0.086407**	**#DIV/0!**	**2.143215**	**1.809818**	**3.564207**
	**CV**	**4.203819**	**9.490978**	**8.404246**	**31.59826**	**#DIV/0!**	**32.57789**	**30.11198**	**28.312**
**81**	**G-OVX**	40	157.8	405.4	0.3973		8.2347	5.1084	13.3431
**82**	**G-OVX**	41	157.4	367.1	0.3773		5.6425	6.7676	12.4101
**83**	**G-OVX**	55	221.66	396.5	0.289		7.3635	5.5815	12.945
**84**	**G-OVX**	58	239	389.1	0.1417		7.8969	6.6373	14.5342
**85**	**G-OVX**	40	165.2	443.2	0.1246		10.9689	10.0076	20.9765
**86**	**G-OVX**	54	196.2	350.1	0.1619		4.5029	5.2576	9.7605
**87**	**G-OVX**	54	215.4	353.4	0.2778		3.9628	3.8774	7.8402
**88**	**G-OVX**	49	217.3	373.8	0.3089		8.569	5.8803	14.4493
**89**	**G-OVX**	40	139.7	327.6	0.164		2.0452	5.0081	7.0533
**90**	**G-OVX**	38	138.80	428	0.2304		7.4789	9.7108	17.1897
**91**	**G-OVX**	36	125.08	415.4	0.22		6.9645	5.6694	12.6339
**92**	**G-OVX**	55	249.6	403.4	0.1504		5.1171	8.9903	14.1074
**93**	**G-OVX**	55	233.8	388.3	0.1397		8.78	10.8812	19.6612
**94**	**G-OVX**	32	104.7	384.5	0.3979		11.6181	9.0918	20.7099
**95**	**G-OVX**	58	227.6	365.9	0.1048		4.4369	5.3972	9.8341
	**AVG**	**47**	**185.9493**	**386.1133**	**0.23238**	**#DIV/0!**	**6.90546**	**6.924433**	**13.82989**
	**SD**	**9.047494**	**46.92498**	**30.91315**	**0.102799**	**#DIV/0!**	**2.635398**	**2.201175**	**4.337946**
	**CV**	**19.24999**	**25.23536**	**8.006237**	**44.23765**	**#DIV/0!**	**38.16397**	**31.78852**	**31.36644**

Fat/BW (%)	Adrenal Gland	Gactroc	stroc/BW (	% mm/fat	eak Mome	ield Mome	ilure Mome	Stiffness	Yield Disp
1.528043	0.0549	1.3208	0.717826	46.97681	259.0137	249.883	244.161	10828.58	0.032071
1.671765	0.0649	1.2201	0.622818	37.33133	201.371	200.193	193.84	10717.38	0.037366
1.764478	0.0742	1.2994	0.661272	37.47693	200.9923	172.003	185.635	10470.6	0.023725
1.847059	0.0416	1.2512	0.618487	33.48499	211.0061	207.472	186.392	8938.459	0.029571
3.069409	0.0249	1.1099	0.612866	19.9669	151.3858	141.33	138.679	5159.144	0.028385
2.258301	0.049	1.1298	0.627318	27.77832	156.3086	145.958	154.331	7183.944	0.02436
2.452358	0.0475	1.3624	0.649071	26.46722	192.4931	182.69	178.65	10762.91	0.020844
1.039269	0.0368	1.0875	0.620719	59.72649	168.2579	163.546	160.6	8076.365	0.037917
0.499194	0.0361	1.0429	0.64696	129.6011	228.1307	200.445	192.703	11797.28	0.019403
0.843815	0.0537	1.4238	0.707303	83.82197	215.4661	197.5	183.657	12124.91	0.02453
0.392747	0.0482	1.099	0.705392	179.6045	192.4931	191.946	165.944	9093.223	0.030588
0.671104	0.0327	1.1803	0.671388	100.0424	210.922	201.329	188.454	10453.26	0.022496
1.206075	0.0276	1.2069	0.637559	52.86234	251.9872	237.177	229.687	12381.04	0.023386
0.522331	0.02342	0.956	0.583282	111.6692	200.2349	190.095	186.603	10286.75	0.022877
**1.411853**	**0.043966**	**1.192143**	**0.648733**	**67.62932**	**202.8616**	**191.5405**	**184.9526**	**9876.702**	**0.026966**
**0.816029**	**0.014991**	**0.131163**	**0.03982**	**47.13761**	**31.30135**	**30.21804**	**27.36939**	**2009.742**	**0.005841**
**57.79839**	**34.09783**	**11.00232**	**6.138087**	**69.69996**	**15.4299**	**15.77632**	**14.79806**	**20.34831**	**21.66009**
0.596177	0.0436	1.2492	0.75801	127.145	219.6736	214.877	193.419	10056.47	0.026351
1.119485	0.0501	1.1095	0.69736	62.29297	202.2125	196.07	187.907	8984.633	0.032113
0.408827	0.0356	0.9233	0.59491	145.5162	181.9744	163.714	176.294	8472.119	0.022073
0.726481	0.0385	1.1532	0.697219	95.97204	187.5703	181.68	171.245	7778.957	0.031901
1.274185	0.0578	1.0305	0.671773	52.72178	183.6153	149.829	153.658	8460.195	0.025208
0.445929	0.0336	1.1441	0.67024	150.3022	217.9485	193.25	186.308	9517.785	0.024318
1.020761	0.0506	1.0482	0.675822	66.20768	193.6291	191.778	182.648	8690.045	0.027029
0.111466	0.0518	1.1854	0.700591	628.526	198.7623	162.914	196.701	8810.268	0.020378
**0.712914**	**0.0452**	**1.105425**	**0.683241**	**166.0855**	**198.1733**	**181.764**	**181.0225**	**8846.309**	**0.026171**
**0.398998**	**0.008699**	**0.102064**	**0.045425**	**190.6963**	**14.52779**	**21.49524**	**13.85763**	**695.7577**	**0.004203**
**55.96722**	**19.2447**	**9.233049**	**6.64848**	**114.8181**	**7.330853**	**11.82591**	**7.655199**	**7.864949**	**16.0582**
1.331738	0.0612	1.3283	0.588785	44.21182	215.0874	196.532	199.351	10248.84	0.023089
1.158051	0.0436	1.2575	0.600812	51.88134	195.3963	166.659	168.342	9852.114	0.020547
0.751669	0.0512	1.1897	0.575568	76.57205	216.3497	201.497	174.106	10576.89	0.02292
1.258295	0.0576	1.4042	0.647097	51.42648	261.4541	247.653	237.303	10288.07	0.033511
1.080018	0.0438	1.3384	0.600988	55.6461	196.1957	177.556	183.784	9942.543	0.019997
1.30762	0.0424	1.4234	0.665451	50.89024	226.9105	226.532	222.408	8871.186	0.033554
1.02431	0.0582	1.2533	0.617998	60.33312	229.2274	191.904	185.214	10239.25	0.021225
1.036369	0.0603	1.1492	0.626609	60.46194	232.2737	214.12	202.423	11397.96	0.028385
1.190029	0.0576	1.3065	0.626318	52.63052	235.3199	251.819	209.239	11008.3	0.02614
0.975554	0.0691	1.3297	0.61333	62.86998	238.3661	156.393	171.329	8523.657	0.020547
0.554448	0.0584	1.2447	0.63635	114.7718	241.4124	163.041	174.527	8976.849	0.02597
0.43274	0.0531	1.2616	0.66087	152.7176	244.4586	155.299	173.812	8256.731	0.025335
0.578821	0.0489	1.0735	0.585972	101.2354	247.5048	174.022	177.557	8305.702	0.022962
0.842082	0.0732	1.4169	0.631136	74.91276	250.551	243.32	219.716	12449.26	0.029783

**0.965839**	**0.055614**	**1.284064**	**0.619806**	**72.18294**	**230.7505**	**197.5962**	**192.7936**	**9924.097**	**0.025283**
**0.292123**	**0.009182**	**0.101574**	**0.027497**	**30.72138**	**19.43826**	**34.29601**	**22.29466**	**1235.985**	**0.004576**
**30.24548**	**16.51029**	**7.910337**	**4.436436**	**42.56045**	**8.423929**	**17.35661**	**11.564**	**12.45439**	**18.09964**
2.773096	0.0726	1.6678	0.564399	20.35268	570.4388	238.646	569.354	31057.3	0.04638
3.180408	0.0687	2.1951	0.64867	20.39582	551.0172	500.728	550.203	42537.38	0.015596
3.134936	0.0898	2.4274	0.651826	20.79233	454.0725	87.885	182.605	41573.12	0.005938
2.823034	0.058	2.0263	0.663925	23.51815	689.1378	676.28	685.836	40068.88	0.031702
2.62119	0.07	1.8671	0.669451	25.53998					
1.340479	0.1068	2.0859	0.703033	52.44644	346.9288	125.1	169.423	20703.31	0.010933
2.029345	0.0727	1.9188	0.747779	36.84827	637.9258	526.171	637.709	47992.75	0.014143
3.52587	0.0694	2.1614	0.665661	18.87933	698.0348	576.949	696.245	51781.03	0.013736
3.645206	0.1303	2.0789	0.617067	16.92819	253.673	99.0063	253.076	37995.24	0.030682
3.609099	0.1156	2.1779	0.623147	17.26601	693.5862	599.734	671.669	58785.33	0.012793
1.809003	0.0314	2.0274	0.780069	43.12149					
2.222982	0.0885	2.4112	0.76328	34.33584	555.5743	534.634	552.807	42607.13	0.015571
5.631438	0.0694	2.2414	0.618659	10.9858	523.0785	445.935	521.994	34656.1	0.019673
4.145618	0.0987	2.1193	0.706198	17.0348	559.3717	439.262	558.829	47895.63	0.011774
4.065042	0.105	2.2866	0.683792	16.82127	707.0945	670.662	705.521	60340.41	0.016157
**3.103783**	**0.083127**	**2.112833**	**0.673797**	**25.01776**	**556.918**	**424.6917**	**519.6362**	**42922.58**	**0.018852**
**1.077993**	**0.025239**	**0.200152**	**0.05908**	**11.53065**	**139.4006**	**214.1511**	**192.3025**	**10882.8**	**0.011018**
**34.73157**	**30.36269**	**9.473171**	**8.768288**	**46.08987**	**25.03072**	**50.42508**	**37.00714**	**25.35449**	**58.44657**
4.420673	0.0767	1.991	0.614317	13.89645	558.6123	549.119	550.419	49137.66	0.013557
3.189118	0.0703	2.0499	0.714998	22.41994	598.3232	597.943	589.969	26850.96	0.027573
4.187272	0.0831	2.3843	0.680451	16.25046	695.2137	642.591	691.525	40420.74	0.032517
3.967899	0.1003	2.3464	0.632965	15.95214	695.8105	564.254	692.61	62732.81	0.011646
2.67291	0.1095	2.4011	0.699417	26.16689	638.414	595.882	636.841	35370.31	0.023114
3.67338	0.1014	2.8356	0.762463	20.75644	1020.226	810.603	866.156	62870.43	0.015799
3.194495	0.0854	2.3508	0.670508	20.98947					
3.319108	0.0596	2.2373	0.656677	19.78476					
3.468296	0.1026	2.1292	0.654736	18.87773	620.7285	582.591	619.481	46653.58	0.02212
2.656446	0.0712	2.1342	0.712826	26.83381	757.2758	729.5	756.432	58630.87	0.01608
3.218521	0.1006	2.25	0.645069	20.0424	680.2407	585.954	678.342	59073.73	0.01213
4.260107	0.0812	2.38	0.638926	14.99789	879.3383	403.891	454.073	66257.26	0.008996
3.649042	0.0701	2.4351	0.706645	19.36523	767.8545	698.631	763.894	65714.96	0.012691
4.292972	0.0804	2.8025	0.716202	16.68314	643.188	461.179	641.669	43093.45	0.017558
**3.583588**	**0.085171**	**2.337671**	**0.679014**	**19.5012**	**712.9354**	**601.8448**	**661.7843**	**51400.56**	**0.017815**
**0.581677**	**0.015287**	**0.246099**	**0.041318**	**3.8639**	**129.5613**	**110.677**	**107.3572**	**13051.04**	**0.00712**
**16.2317**	**17.94841**	**10.52754**	**6.084956**	**19.81365**	**18.17293**	**18.38962**	**16.22239**	**25.39086**	**39.96686**
3.712493	0.0721	2.4487	0.646778	17.42165	804.2755	710.462	794.013	64518.21	0.013694
3.088273	0.0939	1.8666	0.541829	17.54472					
2.746933	0.0476	2.3434	0.697856	25.40491	759.4882	748.389	755.026	71229.64	0.012561
3.934391	0.0592	2.6203	0.62063	15.77449	781.7982	740.134	780.348	78127.26	0.010946
2.830897	0.1317	2.5106	0.652952	23.06519	731.5449	677.666	725.187	75525.86	0.009885
2.995831	0.0749	2.0975	0.620195	20.70194	725.1865	706.279	720.892	73878.53	0.010632

4.978803	0.1087	2.276	0.584789	11.74558	765.8466	644.424	764.731	60030.36	0.012464
3.995925	0.104	2.4102	0.554834	13.885	907.515	859.437	896.583	80854.32	0.014007
4.279337	0.053	2.5427	0.624742	14.59904					
3.630771	0.0754	2.209	0.573617	15.79877	692.8371	677.332	681.459	64962.83	0.012079
2.4698	0.1113	2.5764	0.63725	25.80167	878.6236	859.325	872.711	77671.94	0.013887
2.375398	0.0553	2.4241	0.689448	29.02453	836.9596	819.167	831.382	72341.13	0.014803
2.321633	0.0747	2.7078	0.650132	28.00323	1021.91	959.999	1005.9	97200.47	0.011572
2.928124	0.0911	2.3859	0.661097	22.5775	682.4071	612.019	678.28	64518.46	0.011669
2.986085	0.097	2.4713	0.697516	23.35889	773.5992	770.42	756.811	72796.02	0.020252
**3.28498**	**0.083327**	**2.3927**	**0.630244**	**20.31381**	**797.0762**	**752.6964**	**789.4864**	**73358.08**	**0.012958**
**0.771912**	**0.024569**	**0.214744**	**0.049127**	**5.461446**	**94.73176**	**98.91374**	**92.57183**	**9492.315**	**0.002626**
**23.49822**	**29.48507**	**8.97495**	**7.794846**	**26.88539**	**11.88491**	**13.14125**	**11.72558**	**12.9397**	**20.2681**
3.291342	0.099	2.4902	0.614258	18.66283	915.6024	890.225	907.013	82494.12	0.017744
3.380577	0.1096	2.5707	0.700272	20.71458					
3.264817	0.0512	2.434	0.613871	18.80263	755.4166	746.772	742.477	73451.88	0.011862
3.735338	0.102	2.6932	0.692161	18.53009	838.9676	801.096	824.076	81652.09	0.013019
4.732965	0.0559	2.5685	0.579535	12.24465	966.4134	952.637	953.195	91792.58	0.014297
2.787918	0.0625	2.3476	0.670551	24.05205	775.161	770.643	763.058	63280.94	0.022373
2.218506	0.0741	2.1951	0.621138	27.99801	757.7034	604.768	743.76	72773.1	0.010632
3.865516	0.124	2.3442	0.627127	16.22362	790.8895	747.664	775.105	70751.51	0.013091
2.153022	0.0642	2.4669	0.753022	34.97512					
4.016285	0.0632	2.8567	0.667453	16.61867	974.6123	955.593	947.394	87635.31	0.014176
3.041382	0.0827	2.6264	0.632258	20.78851	766.2369	750.564	761.942	73597.05	0.01596
3.497124	0.0995	2.9636	0.734655	21.00741	906.0091	815.654	903.499	75307.8	0.024471
5.063405	0.0266	2.4325	0.626449	12.37208	818.777	765.679	791.615	77401.05	0.013694
5.38619	0.0596	2.6136	0.67974	12.62005	725.3539	675.268	715.314	86581.08	0.008655
2.687647	0.0702	2.6642	0.728122	27.09145	707.227	676.662	690.662	64468.57	0.012199
**3.541469**	**0.076287**	**2.55116**	**0.662708**	**20.18012**	**822.9515**	**781.0173**	**809.1623**	**77014.39**	**0.014783**
**0.95987**	**0.026029**	**0.199343**	**0.051514**	**6.327161**	**90.18647**	**104.1439**	**89.41821**	**8683.821**	**0.004466**
**27.10371**	**34.11943**	**7.813823**	**7.773196**	**31.35344**	**10.9589**	**13.33439**	**11.05071**	**11.27558**	**30.21332**

Failure Disp	Post-Yield Di	NRG	NRG Post-Yie	Rt. Femur	Normalized Peak Moment	Normalized Stiffness	Peak Momen	Stiffness	Rt. Tibia
0.046687	0.014616	7.4524	3.6917	28.74	262.8322	11236.5	209.3725	12308.26	33.96
0.068039	0.030673	8.8808	6.0361	27.58	193.6857	9896.383			33.74
0.059566	0.035841	8.8433	6.954	27.43	192.727	9587.646	183.0619	11320.49	31.27
0.061261	0.031689	9.554	6.4935	27.4	197.134	7456.535	213.3219	12334.66	33.44
0.055711	0.027326	6.282	3.9882	25.82	158.0077	5866.544	188.0125	11329.36	29.12
0.057617	0.033257	6.9395	5.0842	27.01	163.8972	7994.613	180.1694	9591.938	32.75
0.05766	0.036816	8.8141	6.8739	28.05	171.274	8496.131	192.4069	11115.82	30.38
0.068929	0.031012	7.4117	5.113	27.34	180.5833	9393.057	204.3106	12329.43	33.02
0.049144	0.029741	8.397	6.3248	28.27	244.3519	13195.61	181.5044	9199.133	30.25
0.066429	0.0419	10.8341	8.6862	28	207.3707	11427.05	207.8706	12895.06	31.01
0.058507	0.027919	7.8663	5.1257	26.37	211.9889	10773.83	157.085	7945.699	31.26
0.058549	0.036053	9.6315	7.2651	27	218.2898	11088.38	167.5981	9929.2	30.26
0.040968	0.017582	7.0992	4.2593	27.83	251.1686	12310.47	191.4613	12327.52	30.08
0.034825	0.011947	4.5316	2.3244	27.73	214.8188	11543.94	175.5525	10411.86	32.11
**0.055992**	**0.029026**	**8.038393**	**5.58715**	**27.46929**	**204.8664**	**10019.05**	**188.5944**	**11002.96**	**31.61786**
**0.009952**	**0.008721**	**1.592695**	**1.681565**	**0.757927**	**32.01412**	**2031.76**	**16.93241**	**1496.812**	**1.550911**
**17.77428**	**30.04638**	**19.81359**	**30.09701**	**2.75918**	**15.62683**	**20.27898**	**8.978213**	**13.60373**	**4.905175**
0.039866	0.013515	5.6698	2.7709	26.85	252.4216	10911	209.0944	11048.61	31.08
0.04262	0.010507	4.8917	2.0454	27.1	243.0237	10049.57	180.7813	9725.67	32.67
0.040671	0.018599	5.1805	3.2691	26.95	228.3026	9681.02	187.2337	10923.56	30.75
0.062235	0.030334	8.3636	5.57	27.86	219.4695	8611.345	192.7962	12258.21	33.31
0.062235	0.037028	7.8906	6.1023	27.19	232.4897	9735.539	182.9506	10917.85	32.6
0.068082	0.043764	11.3374	8.9121	27.55	242.3504	10154.53	217.6606	13246.21	31.73
0.042239	0.015209	5.4934	2.8338	26.86	240.0987	9902.637	181.8381	9884.687	32.22
0.046094	0.025716	6.712	4.9183	27.46	225.2861	9502.387	185.7319	11035.75	30.45
**0.050505**	**0.024334**	**6.942375**	**4.552738**	**27.2275**	**235.4303**	**9818.505**	**192.2609**	**11130.07**	**31.85125**
**0.011613**	**0.011958**	**2.181637**	**2.287788**	**0.364564**	**10.90969**	**649.1627**	**13.75186**	**1157.417**	**1.019852**
**22.9932**	**49.14159**	**31.42494**	**50.25081**	**1.338956**	**4.633935**	**6.611625**	**7.152711**	**10.39901**	**3.201922**
0.068802	0.045712	11.6126	9.2653	27.96	228.7506	9200.777	172.7712	10376.36	34.32
0.086977	0.066429	13.6048	11.8494	24.08	203.1442	9257.794	212.4319	13504.74	34.49
0.053508	0.030588	8.6631	6.2578	27.73	223.1541	10054.95	198.5256	12922.89	32.66
0.065031	0.03152	11.7725	7.9726	27.42	271.9963	9479.402	190.905	11197.99	33.42
0.046602	0.026606	6.8921	4.9807	28.13	208.8065	8975.207	191.2944	12762.67	33.28
0.072233	0.03868	12.2213	8.5466	27.85	236.3278	8148.816	193.6863	11202.55	33.75
0.05211	0.030885	8.0717	5.8594	27.33	234.6165	9825.873	186.01	9857.703	31.49
0.055796	0.027411	8.4647	5.7889	27.36	230.6225	11524.62	169.4894	11731.08	30.97
0.057617	0.031478	10.675	7.1593	27.59	242.8138	10433.47	225.8931	12851.41	31.46
0.063167	0.04262	9.6736	7.9258	25.99	248.8358	7720.559	202.92	12023.78	33.36
0.065031	0.039061	9.0739	7.0952	26.82	244.1885	8763.896	189.57	10948.35	32.7
0.063845	0.03851	8.955	7.0183	28.77	245.5291	8174.612	189.7925	9918.27	33.23
0.04584	0.022877	6.2617	4.0855	27.41	245.781	8437.927	196.8569	11788.75	33.49
0.084901	0.055118	16.1684	12.9687	27.01	263.815	11431.82	235.85	11979.15	32.09

**0.062961**	**0.037678**	**10.15074**	**7.626679**	**27.24643**	**237.7416**	**9387.837**	**196.8569**	**11647.55**	**32.90786**
**0.012513**	**0.011907**	**2.70911**	**2.463515**	**1.117921**	**18.72712**	**1169.583**	**18.12747**	**1134.96**	**1.070221**
**19.87351**	**31.60293**	**26.68878**	**32.30128**	**4.103001**	**7.87709**	**12.4585**	**9.208454**	**9.744197**	**3.252173**
-0.00627	-0.00201	18.5668	NaN	33.43	546.3295	33170.63	384.0088	########	39.08
0.019852	0.004256	6.0323	2.2417	33.74	554.7114	42213.56	473.6942	########	40.35
0.006065	0.001127	0.9556	NAN	36.17	479.8021	39317.76	666.5540	########	40.45
0.035117	0.003415	10.6041	2.3169	35	671.3151	41631.16	598.8458	########	41.37
0.010474	-0.00046	1.5585	NAN	34.42	323.5972	22748.47	409.5392	########	39.85
0.019673	0.00553	6.8674	3.2675	32.62	588.6054	52315.99	514.8850	########	38.6
0.019011	0.005275	7.445	3.5655	33.72	692.85	52235.51	571.1440	########	39.79
0.011799	-0.01888	1.1291	NaN	34.33	256.395	37756.64	584.9620	########	39.5
0.026019	0.013226	12.4618	8.6385	34.57	704.4743	57830.92	584.4356	########	38.58
0.02505	0.00948	9.1314	4.9826	33.37	544.6862	43561.54	555.2204	########	40.94
0.029587	0.009913	8.6232	4.7472	34.9	542.2623	32974.52	509.4894	########	39.24
0.018119	0.006345	5.7271	3.2268	33.97	538.2436	49747.64	433.2930	########	39.6
0.021891	0.005734	8.8891	3.9231	34.15	708.1963	60243.84	572.3942	########	39.4
**0.018184**	**0.003304**	**7.5378**	**4.101089**	**34.18385**	**550.1129**	**43519.09**	**527.5743**	**50578.99**	**39.75**
**0.010761**	**0.007877**	**4.890318**	**1.94297**	**0.889068**	**137.887**	**10733.62**	**82.87362**	**11488.24**	**0.84212**
**59.1825**	**238.4513**	**64.87726**	**47.37693**	**2.600843**	**25.06522**	**24.66416**	**15.70843**	**22.71346**	**2.118542**
0.017202	0.003644	5.7663	1.9975	34.07	573.7999	50641.11	382.6928	########	40.07
0.028644	0.00107	8.7752	0.5769	34.48	679.5592	34892.68	482.1824	########	40.25
0.0316	-0.00092	6.6838	NaN	34.47	663.9555	37326.43	489.9468	########	41.1
0.018934	0.007288	7.6403	4.5468	35.15	628.7025	56089.65	688.6628	########	39.51
0.0224	-0.00071	6.0917	NaN	36.37	619.6944	33517.22	427.3710	########	42.61
0.01608	0.00028	15.8898	NaN	35.76	950.9983	56017.48	611.8742	########	42.01
0.028848	0.006728	9.0621	4.0338	34.85	633.9735	47964.73	550.8118	########	40.53
0.019903	0.003823	8.4406	2.8252	34.91	816.0836	64452.38	572.9206	########	39.98
0.020412	0.008282	8.8247	5.2629	35.5	651.8081	56259.13	521.7940	########	41.29
0.008282	-0.00071	4.0223	NaN	35.42	809.0515	59299.42	654.7100	########	41.34
0.020081	0.00739	9.8789	5.3871	34.13	746.8391	63634.61	751.0412	########	40.83
0.026732	0.009174	8.7932	5.373	35.3	539.7004	32849	616.4144	########	41.35
**0.021593**	**0.003778**	**8.322408**	**3.7504**	**35.03417**	**692.8472**	**49411.99**	**562.5352**	**56419.41**	**40.90583**
**0.006537**	**0.003878**	**2.924491**	**1.785401**	**0.686433**	**117.3578**	**11865.98**	**108.5104**	**12977.28**	**0.893191**
**30.27157**	**102.6573**	**35.13997**	**47.60562**	**1.959324**	**16.93849**	**24.01438**	**19.28954**	**23.00144**	**2.18353**
0.020734	0.00704	10.1983	5.3815	33.94	814.2229	########	508.634	50227.15	39.71
				34.49			542.3894	51735.31	40.44
0.013477	0.000916	5.4235	0.6104	34.68	841.5964	78055.43	612.1374	58058.61	42.25
0.017359	0.006413	9.0207	4.821	35.79	718.236	########	431.2532	37835.5	40.83
0.016563	0.006678	8.3295	4.7196	34.54	731.5449	########	556.1416	49033.95	39.69
0.012657	0.002025	5.4138	1.3899	34.23	803.2483	########	600.0302	57465.97	36.16

0.020517	0.008052	9.9626	5.7893	35.45	757.9224	########	532.5194	50033.17	42.05
0.018395	0.004388	9.6229	3.8237	35.46	823.3836	########	515.872	82703.71	43.26
				34.7			586.4096	71050.42	40.47
0.012199	0.000121	4.2525	NaN	36.29	691.8255	########	625.4948	52213.8	41.85
0.015888	0.002001	7.5905	1.6629	35.66	845.2408	########	631.9432	74355.64	41.54
0.017045	0.002242	7.6882	1.8254	34.83	892.429	########	791.7056	49030.08	40.05
0.017624	0.006051	11.632	5.9141	36.82	967.9576	########	716.7594	63057.29	43.92
0.017913	0.006244	7.6983	4.0897	34.98	722.1967	########	548.9694	77584.04	39.8
0.021168	0.000916	5.7783	0.0459	35.74	824.5164	77028.85	424.8048	42714.3	40.93
**0.017041**	**0.004084**	**7.893162**	**3.33945**	**35.17333**	**802.64**	**73820.61**	**575.0043**	**57806.6**	**40.86333**
**0.002926**	**0.002784**	**2.201779**	**2.108356**	**0.799872**	**77.83369**	**8363.775**	**96.36566**	**13241.32**	**1.828534**
**17.16717**	**68.17738**	**27.89477**	**63.13484**	**2.274086**	**9.69721**	**11.32986**	**16.75912**	**22.90624**	**4.474756**

0.020517	0.002773	9.7129	2.4932	35.56	864.8154	77479.38	694.0584	########	41.59
				32.43			757.6212	########	38.03
0.01772	0.005859	8.9368	4.3265	35.3	726.2566	70572.6	600.0960	########	41.21
0.016226	0.003207	7.5346	2.5907	39.05	827.7896	80548.36	756.1736	########	40.49
0.015358	0.001061	7.1905	0.7994	36.33	823.7724	77708.1	736.1704	########	42.22
0.023048	0.000675	7.2922	0.5089	34.68	858.753	71534.87	499.4220	########	40.42
0.019432	0.0088	9.3745	6.2249	34.49	833.2764	80235.24	608.0578	########	40.67
0.023892	0.010801	12.933	8.0543	34.19	816.8905	73318.87	536.2700	########	40.36
				34.36			515.4772	########	40
0.015671	0.001495	7.7926	1.234	35.51	868.9073	77197.92	705.7050	########	42.07
0.019673	0.003713	7.8543	2.7644	36.52	691.1499	66182.9	539.1652	########	41.27
0.031535	0.007064	12.4901	6.3206	35.98	860.0821	70772.94	677.2136	########	39.4
0.018202	0.004508	8.4231	3.5318	33.74	809.543	76489.28	591.4762	########	40.21
0.01109	0.002435	4.7286	1.6014	35.17	725.3539	86581.08	548.4430	########	40.32
0.016708	0.004508	7.3857	3.0809	34.59	752.425	68931.46	549.8906	########	41.56
**0.019159**	**0.004377**	**8.588377**	**3.348538**	**35.19333**	**804.5396**	**75196.38**	**621.016**	**62067.43**	**40.65467**
**0.005018**	**0.003047**	**2.205891**	**2.310212**	**1.496733**	**60.33658**	**5621.489**	**91.5983**	**13038.37**	**1.075891**
**26.19227**	**69.62817**	**25.68461**	**68.99167**	**4.252888**	**7.499517**	**7.475744**	**14.74975**	**21.00677**	**2.646414**

Normalized Peak Moment	Normalized Stiffness
211.974	12326.49
177.4309	11281.03
203.871	12268.43
192.5239	11360.98
185.3394	9628.17
177.9506	11014.51
212.7078	12388.28
199.122	9199.371
199.0783	12894.94
178.259	7945.986
175.6001	9929.308
190.5722	12327.51
191.3918	10412.07
**191.9862**	**10998.24**
**12.85812**	**1494.478**
**6.69742**	**13.58834**
240.9048	11048.7
220.4241	9725.786
232.2355	10923.69
223.7822	12258.3
230.4258	10917.99
241.3638	13246.28
226.9773	9884.819
211.4963	11035.83
**228.4512**	**11130.17**
**10.11311**	**1157.399**
**4.426812**	**10.39875**
157.3874	10376.27
203.7083	13504.69
190.8644	12922.85
179.0352	11197.92
177.0956	12762.58
183.0831	11202.49
179.9423	9857.667
171.3485	11731.09
217.4555	12851.36
191.1319	12023.71
186.4442	10948.33
188.5871	9918.263
198.7978	11788.76
220.9157	11979.06

**188.9855**	**11647.5**
**17.15175**	**1134.954**
**9.075695**	**9.74418**
436.1409	41668
465.7062	55028.11
610.9184	67887.26
637.3843	61802.37
459.9896	44877.86
621.5315	61769.34
582.3552	58710.36
579.0761	62595.89
560.8921	43377.79
578.7639	57930.49
468.008	39613.61
478.9786	43429.58
570.0118	44696.11
**542.289**	**52568.21**
**70.04019**	**9803.976**
**12.91566**	**18.65001**
396.78	35474.9
557.53	56869.8
460.954	40329.1
626.419	68892.8
410.008	42760.6
547.665	50354.9
563.097	62807.7
627.466	61913.7
495.422	46391.6
589.518	62014.2
731.549	70738.3
520.428	51807.9
**543.9029**	**54196.28**
**95.82498**	**11467.54**
**17.61803**	**21.15927**
506.215	50974.03
525.9894	56798.91
592.1704	64223.54
446.7102	33063.06
556.1416	49033.95
581.0472	63327.08

534.4464	49438.19
536.331	76386.87
595.6346	68202.15
625.7408	52137.85
640.0612	71849.16
778.2166	53194.89
729.8794	59006.41
539.2934	80571.56
412.4228	46537.31
**573.3533**	**58316.33**
**95.4696**	**12607.42**
**16.65109**	**21.61901**

660.932	62870
785.2	73586.4
581.076	57504.3
748.883	101687
643.131	59250.6
553.946	52271.4
657.351	53647.7
553.23	52205.9
605.664	62364.9
636.758	64784.1
490.189	44507.6
647.257	62839.3
585.453	63470.3
548.443	55401.8
579.372	65309.3
**618.4589**	**62113.35**
**77.39191**	**13008.65**
**12.51367**	**20.94341**

Animal		Body Wt	Femur Lth	Tibial Lth	T.Ar/length	BV/TV	BS/BV 1/mı	Centroid X,	Centroid y
1	C-FR1	184	28.74	33.96	0.3188365	38.11333	11.1175	3.5006	2.6504
2	C-FR2	195.5	27.58	33.74	0.3920326	33.69499	12.2993	2.4252	2.94199
3	C-FR3	196.5	27.43	31.27	0.3638294	35.06683	11.73619	3.1346	3.12306
4	C-FR4	202.3	27.4	33.44	0.3933102	32.53942	12.47614	2.38574	2.82372
5	C-FR5	181.1	25.82	29.12	0.365294	33.35211	12.67918	2.93225	3.04049
6	C-FR6	180.1	27.01	32.75	0.3609141	34.32743	13.88945	2.6222	2.65259
7	C-FR7	209.9	28.05	30.38					
8	C-FR8	175.2	27.34	33.02	0.338192	33.92275	11.70041	2.88472	2.81831
9	C	161.2	28.27	30.25	0.3033056	44.26298	9.53374	2.65916	3.47518
10	C	201.3	28	31.01	0.3366954	45.05581	9.66097	2.33623	2.60788
11	C	155.8	26.37	31.26	0.3130724	40.75837	9.35979	2.04318	3.01515
12	C	175.8	27	30.26	0.3389637	39.85988	10.47704	3.24916	3.25028
13	C	189.3	27.83	30.08	0.299088	41.57209	9.08167	3.00208	3.29478
14	C	163.9	27.73	32.11	0.2811439	43.19614	9.55475	2.74062	2.98211
	**AVG**	**181.44545**	**27.347273**	**31.243636**	**0.3329979**	**38.884698**	**10.841314**	**2.685534**	**2.996049**
	**SD**	**16.426454**	**0.757927**	**1.5509113**	**0.0351768**	**4.5487241**	**1.5473868**	**0.4089547**	**0.2659431**
	**CV**	**9.0531088**	**2.7714902**	**4.963927**	**10.563674**	**11.69798**	**14.273056**	**15.228059**	**8.8764619**
45	FR-G	164.8	26.85	31.08	0.3472268	38.68055	10.90287	2.46503	2.61504
46	FR-G	159.1	27.1	32.67	0.3105819	40.97594	8.59311	2.76168	2.80279
47	FR-G	155.2	26.95	30.75	0.3476096	35.64067	11.13259	2.71533	2.3327
48	FR-G	165.4	27.86	33.31	0.2870104	40.98974	10.95023	2.38696	2.96071
49	FR-G	153.4	27.19	32.6	0.3290747	35.59106	10.42662	2.47514	2.4173
50	FR-G	170.7	27.55	31.73	0.3568025	36.10696	11.06882	2.42453	2.66568
51	FR-G	155.1	26.86	32.22	0.3026262	36.98663	9.6649	2.51686	3.20387
52	FR-G	169.2	27.46	30.45					
	**AVG**	**161.6125**	**27.2275**	**31.85125**	**0.3258475**	**37.853079**	**10.391306**	**2.5350757**	**2.7140129**
	**SD**	**6.7817902**	**0.3645643**	**1.0198521**	**0.0263941**	**2.3835114**	**0.9438434**	**0.1453904**	**0.3039382**
	**CV**	**4.1963278**	**1.3389563**	**3.2019216**	**8.1001304**	**6.2967439**	**9.0830109**	**5.7351507**	**11.198849**
53	GNRH	225.6	27.96	34.32	0.3526828	39.66233	12.52141	2.68522	3.04975
54	GNRH	209.3	24.08	34.49	0.3749946	43.72736	11.21901	3.54565	2.79389
55	GNRH	206.7	27.73	32.66	0.317524	43.32232	9.92002	2.89083	3.23164
56	GNRH	217	27.42	33.42	0.302078	52.18628	9.80654	2.79622	2.93153
57	GNRH	222.7	28.13	33.28	0.3806722	33.80732	11.77325	2.52205	3.21969
58	GNRH	213.9	27.85	33.75	0.3187228	43.74372	9.06116	2.40299	2.83354
59	GNRH	202.8	27.33	31.49	0.3160666	42.99704	12.49617	2.62982	3.10668
60	GNRH	183.4	27.36	30.97	0.3113063	43.38585	9.59829	2.65889	2.98266
61	GNRH	208.6	27.59	31.46	0.3756818	37.97209	11.45552	3.09429	2.98336
62	GNRH	216.8	25.99	33.36	0.386601	40.73509	14.39917	2.42693	2.81317
63	GNRH	195.6	26.82	32.7	0.3767923	37.8698	11.00245	3.10038	3.00041
64	GNRH	190.9	28.77	33.23	0.3039899	39.62441	10.92155	3.00433	3.12722
65	GNRH	183.2	27.41	33.49	0.3446016	38.85988	11.63692	2.86931	2.29503
66	GNRH	224.61	27.01	32.09					
	**AVG**	**207.22214**	**27.246429**	**32.907857**	**0.3432088**	**41.376422**	**11.216266**	**2.8174546**	**2.9514285**
	**SD**	**14.36789**	**1.1179212**	**1.0702205**	**0.0326782**	**4.4004156**	**1.4456644**	**0.3188246**	**0.2430083**

	**CV**	**6.9335686**	**4.1030009**	**3.2521733**	**9.5213761**	**10.63508**	**12.888999**	**11.316049**	**8.2335812**
		0.0004304	0.5423523	0.016569	0.7446581	0.0769518	0.7712287	0.7070128	0.8149443
**15**	**C-R**	295.5	33.43	39.08	0.2524227	69.27748	5.59883	3.07507	3.13838
**16**	**C-R**	338.4	33.74	40.35	0.3188156	59.81265	12.56165	4.49372	4.39873
**17**	**C-R**	372.4	36.17	40.45					
**18**	**C-R**	305.2	35	41.37	0.259066	67.61805	6.70586	3.69988	3.88554
**19**	**C-R**	278.9							
**20**	**C-R**	296.7	34.42	39.85	0.2838658	63.12123	4.62535	4.43425	4.09252,
**21**	**C-R**	256.6	32.62	38.6	0.2586557	67.64516	4.76055	3.77842	4.17281
**22**	**C-R**	324.7	33.72	39.79	0.2989789	66.69108	4.95405	2.77261	2.44985
**23**	**C-R**	336.9	34.33	39.5					
**24**	**C-R**	349.5	34.57	38.58					
**25**	**C-R**	259.9							
**26**	**C-R**	315.9	33.37	40.94	0.2763659	64.8247	5.53389	3.86609	4.17721
**27**	**C-R**	362.3	34.9	39.24					
**28**	**C-R**	300.1	33.97	39.6	0.2737931	63.69738	5.21445	2.4682	3.01846
**29**	**C-R**	334.4	34.15	39.4	0.2820832	69.74617	5.08307	4.01379	4.20679
	**AVG**	**315.16**	**34.183846**	**39.75**	**0.2782274**	**65.825989**	**6.1153**	**3.6224478**	**3.6809713**
	**SD**	**34.850001**	**0.8890682**	**0.8421203**	**0.0211066**	**3.2318605**	**2.4947394**	**0.7084407**	**0.7142084**
	**CV**	**11.057876**	**2.6008432**	**2.1185417**	**7.5861004**	**4.909703**	**40.795045**	**19.556962**	**19.402716**
**67**	**G-R**	324.1	34.07	40.07	0.2695656	58.64399	7.03162	4.26972	4.06253
**68**	**G-R**	286.7	34.48	40.25	0.2848213	55.61053	7.72899	4.16341	3.79815
**69**	**G-R**	350.4	34.78	41.1	0.2750078	68.89462	4.29409	4.2158	4.38961
**70**	**G-R**	370.7	35.15	39.51	0.2914023	57.73036	6.38828	3.5763	3.83226
**71**	**G-R**	343.3	36.37	42.61	0.2724666	64.08901	6.76535	4.41613	4.05761
**72**	**G-R**	371.9	35.76	42.01	0.3256499	62.16096	4.73481	2.75605	3.19949
**73**	**G-R**	350.6							
**74**	**G-R**	340.7							
**75**	**G-R**	325.2	34.37	40.53					
**76**	**G-R**	299.4	34.91	39.98					
**77**	**G-R**	348.8	35.5	41.29	0.2917679	63.4262	4.95605	3.3134	3.34747
**78**	**G-R**	372.5	35.42	41.34	0.3324994	63.41017	4.44743	4.33813	4.2604
**79**	**G-R**	344.6	34.13	40.83					
**80**	**G-R**	391.3	35.49	41.35	0.3063502	60.81969	4.51831	5.23482	5.19794
	**AVG**	**344.3**	**35.035833**	**40.905833**	**0.2943923**	**61.642837**	**5.6516589**	**4.0315289**	**4.0161622**
	**SD**	**28.663539**	**0.7054136**	**0.893191**	**0.0227829**	**3.9766707**	**1.3183626**	**0.7200438**	**0.5910831**
	**CV**	**8.3251638**	**2.0134061**	**2.1835296**	**7.7389503**	**6.4511481**	**23.327002**	**17.860315**	**14.717611**
		0.0211065	0.0146682	0.0029085	0.1379739	0.0262256	0.6287489	0.2420136	0.3063978
**30**	**C-OVX**	378.6	33.94	39.71	0.303576	66.64111	4.96486	5.37806	5.35385
**31**	**C-OVX**	344.5	34.49	40.44	0.2907765	65.23805	6.82532	3.54714	3.89632
**32**	**C-OVX**	335.8	34.68	42.25	0.2720003	72.35654	4.46965	4.62967	4.83183
**33**	**C-OVX**	422.2	35.79	40.83	0.3067779	67.79909	4.76772	4.79393	4.7172
**34**	**C-OVX**	384.5	34.54	39.69					
**35**	**C-OVX**	338.2	34.23	36.16	0.2658516	70.72435	5.48723	3.36303	3.90071
**36**	**C-OVX**	389.2	35.45	42.05	0.3061896	56.6815	6.57801	3.85488	4.51774
**37**	**C-OVX**	434.4	35.46	43.26	0.3372363	53.87879	8.69907	3.95513	3.90794

38	C-OVX	407	34.7	40.47					
39	C-OVX	385.1	36.29	41.85					
40	C-OVX	404.3	35.66	41.54					
41	C-OVX	351.6	34.83	40.05					
42	C-OVX	416.5	36.82	43.92					
43	C-OVX	360.9	34.98	39.8					
44	C-OVX	354.3	35.74	40.93					
	**AVG**	**380.47333**	**35.173333**	**40.863333**	**0.2974869**	**64.759919**	**5.9702657**	**4.2174057**	**4.4465129**
	**SD**	**31.975917**	**0.799872**	**1.8285344**	**0.0240643**	**6.9515821**	**1.4993872**	**0.7335857**	**0.5687338**
	**CV**	**8.4042464**	**2.2740865**	**4.4747559**	**8.0891965**	**10.73439**	**25.114246**	**17.394241**	**12.790557**
81	G-OVX	405.4	35.56	41.59	0.3128099	67.70145	4.57407	5.4196	5.34408
82	G-OVX	367.1	32.43	38.03	0.3211363	70.52253	4.16967	3.33381	3.70069
83	G-OVX	396.5	35.3	41.21					
84	G-OVX	389.1	39.05	40.49	0.2681344	66.60042	5.36385	3.55731	4.6269
85	G-OVX	443.2	36.33	42.22					
86	G-OVX	350.1	34.68	40.42	0.282526	65.8936	4.53545	4.03588	3.9262
87	G-OVX	353.4	34.49	40.67	0.3295309	58.43572	5.04994	3.11725	4.13784
88	G-OVX	373.8	34.19	40.36	0.331993	62.11715	4.4487	3.98278	4.85069
89	G-OVX	327.6	34.36	40					
90	G-OVX	428	35.51	42.07	0.2943399	67.07606	4.82341	3.90225	4.05647
91	G-OVX	415.4	36.52	41.27	0.2813264	63.11891	4.70688	3.96236	4.49958
92	G-OVX	403.4	35.98	39.4					
93	G-OVX	388.3	33.74	40.21	0.276048	68.53787	4.34097	4.13624	4.10507
94	G-OVX	384.5	35.17	40.32	0.2669963	62.16369	5.2667	4.03351	4.24595
95	G-OVX	365.9	34.59	41.56					
	**AVG**	**386.11333**	**35.193333**	**40.654667**	**0.2964841**	**65.21674**	**4.727964**	**3.948099**	**4.349347**
	**SD**	**30.913147**	**1.496733**	**1.0758908**	**0.0252703**	**3.659031**	**0.3955306**	**0.6182051**	**0.4877531**
	**CV**	**8.0062366**	**4.2528877**	**2.646414**	**8.5233164**	**5.6105703**	**8.3657711**	**15.658298**	**11.214398**
						0.8617136	0.0231143	0.4252743	0.7107501

Centroid z	T.Ar	B.Ar	Ma.Ar	RCA	RMA	T.Pm	B.Pm	Ec.PM	Ec.Pm/T.PM
3.15377	9.16336	3.50101	5.66235	0.3820662	0.6179338	12.2299	32.59171	20.36181	1.6649204
4.85931	10.81226	3.65266	7.1596	0.3378258	0.6621742	12.95147	37.39786	24.44639	1.8875379
4.08553	9.97984	3.5082	6.47164	0.3515287	0.6484713	12.81974	34.73354	21.9138	1.7093794
3.70974	10.7767	3.51477	7.26193	0.3261453	0.6738547	12.89375	37.01511	24.12136	1.8707793
3.10937	9.43189	3.15457	6.27732	0.3344579	0.6655421	12.04448	33.97559	21.93111	1.8208432
3.45306	9.74829	3.35708	6.39121	0.3443763	0.6556237	12.21386	39.0852	26.87134	2.2000694
4.04842	9.24617	3.14417	6.102	0.3400511	0.6599489	11.83316	30.81014	18.97698	1.6037119
4.25191	8.57445	3.80331	4.77114	0.4435631	0.5564369	12.20148	30.45578	18.2543	1.4960726
1.51323	9.42747	4.25619	5.17128	0.4514668	0.5485332	12.15754	34.48491	22.32737	1.8365039
3.0126	8.25572	3.3713	4.88442	0.4083593	0.5916407	11.17217	26.76224	15.59007	1.3954379
3.03323	9.15202	3.65602	5.496	0.3994768	0.6005232	11.82435	32.47122	20.64687	1.7461315
3.70846	8.32362	3.46706	4.85656	0.4165327	0.5834673	11.17654	26.60123	15.42469	1.3800953
3.38669	7.79612	3.37437	4.42175	0.4328268	0.5671732	10.80052	27.04697	16.24645	1.5042285
**3.322671**	**9.073245**	**3.509884**	**5.563361**	**0.3897256**	**0.6102744**	**11.831785**	**31.870839**	**20.039054**	**1.6853874**
**0.8078778**	**0.9145256**	**0.2895859**	**0.9256245**	**0.0454848**	**0.0454848**	**0.6705125**	**4.1157818**	**3.5590476**	**0.2300998**
**24.314108**	**10.079367**	**8.2505843**	**16.637865**	**11.670971**	**7.453166**	**5.6670439**	**12.913942**	**17.760557**	**13.652634**
3.43814	9.32304	3.61466	5.70838	0.3877126	0.6122874	11.80786	33.21255	21.40469	1.8127493
3.15859	8.41677	3.45491	4.96186	0.4104793	0.5895207	11.34472	25.08552	13.7408	1.2112066
3.31622	9.36808	3.34629	6.02179	0.3572013	0.6427987	11.98611	31.66092	19.67481	1.6414675
2.26691	7.99611	3.28559	4.71052	0.4108985	0.5891015	11.02163	30.50407	19.48244	1.7676551
3.5851	8.94754	3.1916	5.75594	0.3567014	0.6432986	11.5939	28.28011	16.68621	1.4392232
3.69188	9.82991	3.55756	6.27235	0.3619118	0.6380882	12.14638	33.50154	21.35516	1.7581502
3.29924	8.12854	3.01277	5.11577	0.370641	0.629359	11.00981	24.78048	13.77067	1.2507636
**3.2508686**	**8.85857**	**3.3519114**	**5.5066586**	**0.3793637**	**0.6206363**	**11.55863**	**29.575027**	**18.016397**	**1.5544594**
**0.4698533**	**0.6950681**	**0.2109833**	**0.5827968**	**0.0238668**	**0.0238668**	**0.4521944**	**3.6213099**	**3.3066551**	**0.2531664**
**14.453162**	**7.8462791**	**6.2944167**	**10.583492**	**6.2912692**	**3.8455357**	**3.9121799**	**12.244485**	**18.353587**	**16.28646**
3.43917	9.86101	3.92175	5.93926	0.3977027	0.6022973	12.1714	40.97555	28.80415	2.3665437
3.06937	9.02987	3.95804	5.07183	0.4383275	0.5616725	11.86897	37.34642	25.47745	2.1465595
2.87674	8.80494	3.82277	4.98217	0.434162	0.565838	11.65197	31.50982	19.85785	1.7042483
2.61072	8.28298	4.33137	3.95161	0.5229241	0.4770759	11.25423	35.00254	23.74831	2.1101675
3.43753	10.70831	3.62912	7.07919	0.3389069	0.6610931	12.88758	36.53083	23.64325	1.8345764
2.59176	8.87643	3.8897	4.98673	0.4382054	0.5617946	11.65023	29.3879	17.73767	1.5225167
3.17559	8.6381	3.72374	4.91436	0.4310832	0.5689168	11.54585	38.99386	27.44801	2.3773053
3.03618	8.51734	3.7029	4.81444	0.4347484	0.5652516	11.33564	29.82408	18.48844	1.631001
4.69818	10.36506	3.9458	6.41926	0.3806828	0.6193172	12.57662	37.70095	25.12433	1.9977013
4.07154	10.04776	4.10498	5.94278	0.4085468	0.5914532	12.93877	49.36367		
2.8689	10.10557	3.83623	6.26934	0.3796154	0.6203846	12.40061	35.7036	23.30299	1.8791809
2.47169	8.74579	3.47368	5.27211	0.3971831	0.6028169	11.45691	32.14285	20.68594	1.8055427
2.89728	9.44553	3.67938	5.76615	0.3895366	0.6104634	12.00169	36.17316	24.17147	2.0140055
**3.1726654**	**9.3406685**	**3.8476508**	**5.4930177**	**0.4147404**	**0.5852596**	**11.980036**	**36.204248**	**23.207488**	**1.9491124**
**0.626228**	**0.7921385**	**0.2202766**	**0.8350302**	**0.0440377**	**0.0440377**	**0.5734141**	**5.2817803**	**3.4287425**	**0.271503**

**19.738229**	**8.4805334**	**5.7249644**	**15.201666**	**10.618135**	**7.5244715**	**4.7864138**	**14.588841**	**14.774294**	**13.929569**
0.2791694	0.8665806	0.0034322	0.4414669	0.0762394	0.0762394	0.8572237	0.0622002	0.0700159	0.0213779
4.87908	8.43849	5.84597	2.59252	0.6927744	0.3072256	11.58884	20.59644	9.0076	0.777265
7.07019	10.75684	6.43395	4.32289	0.5981264	0.4018736	13.60378	56.38346	42.77968	3.1446907
5.03817	9.06731	6.13114	2.93617	0.6761807	0.3238193	11.87618	28.305	16.42882	1.3833421
18.19217	9.77066	6.16736	3.6033	0.6312122	0.3687878	12.3555	20.44678	8.09128	
17.42864	8.43735	5.70746	2.72989	0.6764517	0.3235483	11.61072	19.32138	7.71066	
3.89317	10.08157	6.72351	3.35806	0.666911	0.333089	12.39088	23.16257	10.77169	
4.78727	9.22233	5.97834	3.24399	0.6482462	0.3517538	11.88249	22.96019	11.0777	
5.69004	9.30075	5.92434	3.37641	0.6369744	0.3630256	11.96059	21.7568	9.79621	
18.72603	9.63314	6.71875	2.91439	0.6974621	0.3025379	12.43633	23.71104	11.27471	
**9.5227511**	**9.4120489**	**6.1812022**	**3.2308467**	**0.6582599**	**0.3417401**	**12.189479**	**26.29374**	**14.104261**	**1.7684326**
**6.5082552**	**0.7499404**	**0.369726**	**0.5260968**	**0.0323187**	**0.0323187**	**0.619458**	**11.580519**	**11.053987**	**1.2297955**
**68.344275**	**7.9678766**	**5.981457**	**16.283557**	**4.9097104**	**9.4570862**	**5.0819073**	**44.042874**	**78.373386**	**69.541555**
5.14791	9.1841	5.38592	3.79818	0.5864396	0.4135604	11.97107	26.65923	14.68816	
5.11603	9.82064	5.46131	4.35933	0.5561053	0.4438947	12.41153	29.54638	17.13485	
19.69539	9.56477	6.58961	2.97516	0.688946	0.311054	12.32779	20.17953	7.85174	
4.96923	10.24279	5.9132	4.32959	0.5773036	0.4226964	12.56013	26.40783	13.8477	
5.10006	9.90961	6.35097	3.55864	0.64089	0.35911	12.54797	29.52022	16.97225	
5.64675	11.64524	7.2388	4.40644	0.6216102	0.3783898	13.35588	23.96522	10.60934	
6.30012	10.35776	6.56953	3.78823	0.6342617	0.3657383	12.74664	22.93457	10.18793	
19.24006	11.77713	7.4679	4.30923	0.6341019	0.3658981	13.74037	23.13019	9.38982	
18.65576	10.87237	6.61254	4.25983	0.6081967	0.3918033	12.90023	21.68123	8.781	
**9.9857011**	**10.374934**	**6.3988644**	**3.97607**	**0.6164283**	**0.3835717**	**12.729068**	**24.8916**	**12.162532**	**#DIV/0!**
**6.925077**	**0.8974443**	**0.7161289**	**0.4862607**	**0.0397667**	**0.0397667**	**0.5411772**	**3.3319544**	**3.5548031**	**#DIV/0!**
**69.349933**	**8.6501201**	**11.1915**	**12.229681**	**6.4511437**	**10.36747**	**4.251507**	**13.385859**	**29.227491**	**#DIV/0!**
0.885634	0.0251491	0.4297013	0.0065878	0.0262255	0.0262255	0.0666522	0.7315875	0.6227299	#DIV/0!
21.99672	10.30337	6.86628	3.43709	0.6664111	0.3335889	12.9494	23.54516	10.59576	
2.42151	10.02888	6.54264	3.48624	0.6523799	0.3476201	12.3734	30.60048	18.22708	
19.17569	9.43297	6.82537	2.6076	0.7235653	0.2764347	12.2297	21.09064	8.86094	
21.79823	10.97958	7.44406	3.53552	0.6779913	0.3220087	13.34749	24.14096	10.79347	
4.57506	9.1001	6.43598	2.66412	0.7072428	0.2927572	12.033	23.51929	11.48629	
5.13527	10.85442	6.15245	4.70197	0.5668152	0.4331848	12.98168	28.88667	15.90499	
5.07882	11.9584	6.44304	5.51536	0.5387878	0.4612122	13.62767	39.22543	25.59776	

**11.454471**	**10.379674**	**6.6728314**	**3.7068429**	**0.6475991**	**0.3524009**	**12.791763**	**27.286947**	**14.495184**	**#DIV/0!**
**9.0115805**	**0.9781005**	**0.4187547**	**1.057308**	**0.0695157**	**0.0695157**	**0.5965393**	**6.225668**	**5.9003974**	**#DIV/0!**
**78.673036**	**9.4232293**	**6.2755181**	**28.523142**	**10.734376**	**19.726315**	**4.6634641**	**22.815554**	**40.705915**	**#DIV/0!**
18.77774	11.12352	7.53079	3.59273	0.677015	0.322985	13.19406	23.73259	10.53853	
4.49348	10.41445	7.34453	3.06992	0.705225	0.294775	12.62624	21.47467	8.84843	
4.56316	10.47065	6.9735	3.49715	0.6660045	0.3339955	13.2702	25.28077	12.01057	
3.78751	9.798	6.45626	3.34174	0.6589365	0.3410635	12.46563	20.81819	8.35256	
5.01204	11.36552	6.64153	4.72399	0.5843578	0.4156422	13.46246	24.14718	10.68472	
19.29129	11.35084	7.05082	4.30002	0.6211716	0.3788284	13.2984	22.33789	9.03949	
4.90569	10.45201	7.01079	3.44122	0.67076	0.32924	12.88009	23.53018	10.65009	
5.16455	10.27404	6.48486	3.78918	0.6311889	0.3688111	12.86481	21.89281	9.028	
4.10507	9.31386	6.38352	2.93034	0.6853786	0.3146214	11.98147	19.55653	7.57506	
5.04735	9.39026	5.83733	3.55293	0.6216367	0.3783633	12.10457	22.29282	10.18825	
**7.514788**	**10.395315**	**6.771393**	**3.623922**	**0.6521675**	**0.3478325**	**12.814793**	**22.506363**	**9.69157**	**#DIV/0!**
**6.0880784**	**0.741248**	**0.5058108**	**0.5393034**	**0.0365902**	**0.0365902**	**0.5129071**	**1.6999253**	**1.3373224**	**#DIV/0!**
**81.014639**	**7.1305968**	**7.4698188**	**14.88176**	**5.610548**	**10.519478**	**4.0024614**	**7.5530873**	**13.798821**	**#DIV/0!**
0.2969018	0.9704991	0.6783608	0.833883	0.861708	0.861708	0.9331548	0.0337505	0.0237022	

MMI(polar)	Ct.Th	Rt. Femur	T.Ar/Le
8.49889	0.21484	28.74	0.318836
10.95825	0.19534	27.58	0.392033
9.81084	0.20201	27.43	0.363829
10.55716	0.18991	27.4	0.39331
8.21713	0.1857	25.82	0.365294
8.7563	0.17178	27.01	0.360914
		28.05	
7.9345	0.2041	27.34	0.338192
8.30552	0.24976	28.27	0.303306
10.25458	0.24298	28	0.336695
7.27179	0.25194	26.37	0.313072
8.76109	0.22519	27	0.338964
7.41733	0.26067	27.83	0.299088
6.66449	0.24952	27.73	0.281144
**8.413989**	**0.223155**	**27.46929**	**0.338821**
**1.3242159**	**0.0296988**	**0.757927**	**0.035177**
**15.738265**	**13.308605**	**2.75918**	**10.38211**
8.78601	0.21767	26.85	0.347227
7.68957	0.27545	27.1	0.310582
8.47038	0.21138	26.95	0.34761
6.88957	0.21542	27.86	0.28701
7.65164	0.22571	27.19	0.329075
9.29957	0.21238	27.55	0.356803
6.48586	0.24316	26.86	0.302626
		27.46	
**7.8960857**	**0.2287386**	**27.2275**	**0.325847**
**1.0161824**	**0.0233378**	**0.364564**	**0.026394**
**12.869445**	**10.202843**	**1.338956**	**8.10013**
9.93704	0.19142	27.96	0.352683
9.19889	0.21196	24.08	0.374995
8.7257	0.24264	27.73	0.317524
8.55526	0.24749	27.42	0.302078
10.70296	0.19869	28.13	0.380672
8.8393	0.26471	27.85	0.318723
8.39004	0.19099	27.33	0.316067
8.1014	0.24832	27.36	0.311306
10.70153	0.20932	27.59	0.375682
10.81955	0.16632	25.99	0.386601
10.28837	0.21489	26.82	0.376792
7.82346	0.21614	28.77	0.30399
9.19633	0.20343	27.41	0.344602
		27.01	
**9.3292177**	**0.2158708**	**27.24643**	**0.343209**
**1.047844**	**0.0278926**	**1.117921**	**0.032678**

**11.231853**	**12.920964**	**4.103001**	**9.521376**
0.2083461	0.8011035		
10.97666	0.56767	33.43	0.252423
16.44009	0.22822	33.74	0.318816
		36.17	
12.50311	0.43322	35	0.259066
13.7103	0.60326	34.42	0.283866
10.64674	0.59079	32.62	0.258656
14.75526	0.58055	33.72	0.298979
		34.33	
		34.57	
12.61304	0.52076	33.37	0.276366
		34.9	
12.38708	0.5446	33.97	0.273793
13.86623	0.56672	34.15	0.282083
**13.099834**	**0.5150878**	**34.18385**	**0.278227**
**1.819491**	**0.1189117**	**0.889068**	**0.021107**
**13.88942**	**23.085706**	**2.600843**	**7.5861**
11.96072	0.40406	34.07	0.269566
13.20706	0.36968	34.48	0.284821
14.02396	0.6531	34.47	0.277481
14.43043	0.44784	35.15	0.291402
14.81336	0.43028	36.37	0.272467
19.55814	0.60411	35.76	0.32565
		34.85	
		34.91	
15.86571	0.57289	35.5	0.291768
20.09365	0.64573	35.42	0.332499
		34.13	
16.59697	0.60998	35.3	0.307999
**15.616667**	**0.5264078**	**35.03417**	**0.29485**
**2.7468831**	**0.1120364**	**0.686433**	**0.022647**
**17.589433**	**21.283201**	**1.959324**	**7.680734**
0.0358308	0.8379591		
16.18305	0.58324	33.94	0.303576
14.64799	0.42762	34.49	0.290776
13.84508	0.64724	34.68	0.272
18.29431	0.61672	35.79	0.306778
		34.54	
12.72111	0.54729	34.23	0.265852
16.09757	0.42597	35.45	0.30619
18.91727	0.32851	35.46	0.337236

		34.7	
		36.29	
		35.66	
		34.83	
		36.82	
		34.98	
		35.74	
**15.815197**	**0.5109414**	**35.17333**	**0.297487**
**2.2657234**	**0.1181514**	**0.799872**	**0.024064**
**14.326242**	**23.124258**	**2.274086**	**8.089196**
18.34591	0.63464	35.56	0.31281
16.40696	0.68402	32.43	0.321136
		35.3	
16.79677	0.55168	39.05	0.268134
		36.33	
14.28752	0.62025	34.68	0.282526
17.41542	0.55009	34.49	0.329531
18.33123	0.63129	34.19	0.331993
		34.36	
16.90957	0.5959	35.51	0.29434
15.65339	0.59242	36.52	0.281326
		35.98	
12.86126	0.65283	33.74	0.276048
12.79791	0.5237	35.17	0.266996
		34.59	
**15.980594**	**0.603682**	**35.19333**	**0.296484**
**2.0473057**	**0.0505807**	**1.496733**	**0.02527**
**12.811199**	**8.3786951**	**4.252888**	**8.523316**
0.8773173	0.041415		

Animal		Body Wt	Femur Lth	Tibial Lth	BV/TV	SMI	Tb.Th	Tb.N	Tb.Sp
**1**	**C-FR1**	184	28.74	33.96	7.38893	1.3853	0.06146	1.20221	0.75874
**2**	**C-FR2**	195.5	27.58	33.74	9.56411	1.18973	0.06385	1.49785	0.70785
**3**	**C-FR3**	196.5	27.43	31.27	7.71605	1.29386	0.05851	1.31877	0.6892
**4**	**C-FR4**	202.3	27.4	33.44	5.78578	1.65482	0.05673	1.01981	0.77038
**5**	**C-FR5**	181.1	25.82	29.12	7.68835	1.37497	0.05861	1.31177	0.65975
**6**	**C-FR6**	180.1	27.01	32.75	8.21049	1.39799	0.05402	1.52	0.56302
**7**	**C-FR7**	209.9	28.05	30.38					
**8**	**C-FR8**	175.2	27.34	33.02	5.89602	1.60924	0.05654	1.04285	0.68313
**9**	**C**	161.2	28.27	30.25	6.93181	1.60526	0.06245	1.11004	0.70216
**10**	**C**	201.3	28	31.01	8.66396	1.29144	0.06421	1.34925	0.68312
**11**	**C**	155.8	26.37	31.26	4.35361	1.88994	0.0599	0.72683	0.75754
**12**	**C**	175.8	27	30.26	7.49156	1.54322	0.05823	1.28661	0.65069
**13**	**C**	189.3	27.83	30.08	9.75662	1.37846	0.05876	1.66056	0.52941
**14**	**C**	163.9	27.73	32.11	7.56263	1.36088	0.0574	1.3175	0.62158
	**AVG**	**181.4455**	**27.34727**	**31.24364**	**7.234083**	**1.510622**	**0.058685**	**1.234522**	**0.662078**
	**SD**	**17.74454**	**0.753367**	**1.400666**	**1.564118**	**0.183617**	**0.002948**	**0.268291**	**0.076393**
	**CV**	**9.779545**	**2.754816**	**4.483044**	**21.62152**	**12.15507**	**5.023928**	**21.73238**	**11.5384**
**45**	**FR-G**	164.8	26.85	31.08	4.20619	1.66484	0.06098	0.68975	0.91587
**46**	**FR-G**	159.1	27.1	32.67	4.33633	1.54547	0.05982	0.72492	0.99709
**47**	**FR-G**	155.2	26.95	30.75	2.76147	1.76761	0.05567	0.49608	1.09968
**48**	**FR-G**	165.4	27.86	33.31	5.31477	1.6311	0.05916	0.89843	0.6653
**49**	**FR-G**	153.4	27.19	32.6	3.61468	1.78897	0.0577	0.62645	0.87931
**50**	**FR-G**	170.7	27.55	31.73	6.06099	1.64782	0.06047	1.00229	0.62366
**51**	**FR-G**	155.1	26.86	32.22	3.23193	1.76042	0.05848	0.55269	0.99608
**52**	**FR-G**	169.2	27.46	30.45					
	**AVG**	**161.6125**	**27.2275**	**31.85125**	**4.218051**	**1.686604**	**0.058897**	**0.712944**	**0.882427**
	**SD**	**6.78179**	**0.364564**	**1.019852**	**1.159847**	**0.088945**	**0.001815**	**0.182117**	**0.177246**
	**CV**	**4.196328**	**1.338956**	**3.201922**	**27.49722**	**5.273595**	**3.080801**	**25.54438**	**20.08615**
**53**	**GNRH**	225.6	27.96	34.32	4.08592	1.96553	0.05647	0.72358	0.70369
**54**	**GNRH**	209.3	24.08	34.49	6.36269	1.49227	0.05664	1.12342	0.77242
**55**	**GNRH**	206.7	27.73	32.66	4.85196	1.59383	0.05806	0.83567	0.78569
**56**	**GNRH**	217	27.42	33.42	4.85752	1.68049	0.05664	0.85757	0.74292
**57**	**GNRH**	222.7	28.13	33.28	5.73986	1.55357	0.05361	1.07071	0.68442
**58**	**GNRH**	213.9	27.85	33.75	3.42708	1.75447	0.05576	0.61461	0.81264
**59**	**GNRH**	202.8	27.33	31.49	6.39319	1.4349	0.05608	1.1401	0.6395
**60**	**GNRH**	183.4	27.36	30.97	5.257	1.6892	0.058	0.90635	0.73269
**61**	**GNRH**	208.6	27.59	31.46	7.91684	1.5095	0.06017	1.31569	0.67501
**62**	**GNRH**	216.8	25.99	33.36	3.9163	1.81216	0.0606	0.64622	0.808
**63**	**GNRH**	195.6	26.82	32.7	3.72161	1.84256	0.05981	0.62221	0.90309
**64**	**GNRH**	190.9	28.77	33.23	4.08072	1.84899	0.05523	0.73881	0.71942
**65**	**GNRH**	183.2	27.41	33.49	4.15778	1.82986	0.05554	0.74854	0.74917
**66**	**GNRH**	224.61	27.01	32.09					
	**AVG**	**207.2221**	**27.24643**	**32.90786**	**4.98219**	**1.692872**	**0.057124**	**0.872575**	**0.748358**
	**SD**	**14.36789**	**1.117921**	**1.070221**	**1.311173**	**0.165254**	**0.002092**	**0.225235**	**0.06954**

	**CV**	**6.933569**	**4.103001**	**3.252173**	**26.31721**	**9.761785**	**3.661951**	**25.81271**	**9.292392**
		0.00043	0.542352	0.016569	0.000153	0.002764	0.043814	0.000327	0.014508
**15**	**C-R**	295.5	33.43	39.08	35.1	2.167	0.08625	4.07	0.15669
**16**	**C-R**	338.4	33.74	40.35	34.1736	1.6689	0.09158	3.73136	0.17985
**17**	**C-R**	372.4	36.17	40.45					
**18**	**C-R**	305.2	35	41.37	23.9266	1.63046	0.0735	3.2546	0.2149
**19**	**C-R**	278.9							
**20**	**C-R**	296.7	34.42	39.85	25.252	1.2697	0.077	3.274	0.218
**21**	**C-R**	256.6	32.62	38.6	20.3533	1.699	0.073	2.782	0.23188
**22**	**C-R**	324.7	33.72	39.79	26.1735	1.449	0.08	3.237	0.216
**23**	**C-R**	336.9	34.33	39.5					
**24**	**C-R**	349.5	34.57	38.58					
**25**	**C-R**	259.9							
**26**	**C-R**	315.9	33.37	40.94	22.0679	1.3957	0.082	2.69083	0.27246
**27**	**C-R**	362.3	34.9	39.24					
**28**	**C-R**	300.1	33.97	39.6	21.2666	1.783	0.078	2.722	0.24861
**29**	**C-R**	334.4	34.15	39.4	24.528	1.36	0.08207	2.988	0.25086
	**AVG**	**315.16**	**34.18385**	**39.75**	**25.87128**	**1.602529**	**0.080378**	**3.194421**	**0.221028**
	**SD**	**34.85**	**0.889068**	**0.84212**	**5.318314**	**0.274234**	**0.005962**	**0.468534**	**0.035918**
	**CV**	**11.05788**	**2.600843**	**2.118542**	**20.55683**	**17.11257**	**7.41807**	**14.66727**	**16.25038**
**67**	**G-R**	324.1	34.07	40.07	20.39	1.727	0.0761	2.6799	0.2813
**68**	**G-R**	286.7	34.48	40.25	16.2	1.77	0.074	2.1892	0.3383
**69**	**G-R**	350.4	34.78	41.1	19.647	1.86	0.079	2.47	0.257
**70**	**G-R**	370.7	35.15	39.51	17.232	1.7229	0.0713	2.4164	0.2773
**71**	**G-R**	343.3	36.37	42.61	22.0763	1.44149	0.0746	2.958	0.2575
**72**	**G-R**	371.9	35.76	42.01	16.01	1.81	0.075	2.125	0.319
**73**	**G-R**	350.6							
**74**	**G-R**	340.7							
**75**	**G-R**	325.2	34.37	40.53					
**76**	**G-R**	299.4	34.91	39.98					
**77**	**G-R**	348.8	35.5	41.29	16.942	1.77	0.0729	2.32	0.3046
**78**	**G-R**	372.5	35.42	41.34	19.05	1.63	0.085	2.238	0.3
**79**	**G-R**	344.6	34.13	40.83					
**80**	**G-R**	391.3	35.49	41.35	19.358	1.58	0.0769	2.517	0.266
	**AVG**	**344.3**	**35.03583**	**40.90583**	**18.54503**	**1.701266**	**0.076089**	**2.434833**	**0.289**
	**SD**	**28.66354**	**0.705414**	**0.893191**	**2.068312**	**0.12984**	**0.004022**	**0.262286**	**0.028364**
	**CV**	**8.325164**	**2.013406**	**2.18353**	**11.15292**	**7.631937**	**5.285862**	**10.77223**	**9.814646**
		0.021106	0.014668	0.002908	0.00141	0.343474	0.092564	0.000619	0.000399
**30**	**C-OVX**	378.6	33.94	39.71	12.3128	2.1734	0.07	1.7579	0.2893
**31**	**C-OVX**	344.5	34.49	40.44	16.03	1.81	0.06897	2.32	0.26645
**32**	**C-OVX**	335.8	34.68	42.25	16.799	1.7866	0.0729	2.3025	0.2636
**33**	**C-OVX**	422.2	35.79	40.83	7.9849	2.3268	0.0759	1.0517	0.4246
**34**	**C-OVX**	384.5	34.54	39.69					
**35**	**C-OVX**	338.2	34.23	36.16	13.944	2.16	0.073	1.909	0.337
**36**	**C-OVX**	389.2	35.45	42.05	9.6116	2.004	0.0646	1.48741	0.3713
**37**	**C-OVX**	434.4	35.46	43.26	17.2276	1.757	0.0751	2.2924	0.2734

**38**	**C-OVX**	407	34.7	40.47					
**39**	**C-OVX**	385.1	36.29	41.85					
**40**	**C-OVX**	404.3	35.66	41.54					
**41**	**C-OVX**	351.6	34.83	40.05					
**42**	**C-OVX**	416.5	36.82	43.92					
**43**	**C-OVX**	360.9	34.98	39.8	9.098	1.9041	0.0739	1.2308	0.62566
**44**	**C-OVX**	354.3	35.74	40.93					
	**AVG**	**380.4733**	**35.17333**	**40.86333**	**12.87599**	**1.990238**	**0.071796**	**1.793964**	**0.356414**
	**SD**	**31.97592**	**0.799872**	**1.828534**	**3.675874**	**0.210986**	**0.003739**	**0.501481**	**0.122966**
	**CV**	**8.404246**	**2.274086**	**4.474756**	**28.54829**	**10.60103**	**5.207472**	**27.95378**	**34.50097**
**81**	**G-OVX**	405.4	35.56	41.59	14.726	1.8932	0.0696	2.1151	0.2684
**82**	**G-OVX**	367.1	32.43	38.03	9.419	2.189	0.073	1.2767	0.44431
**83**	**G-OVX**	396.5	35.3	41.21					
**84**	**G-OVX**	389.1	39.05	40.49					
**85**	**G-OVX**	443.2	36.33	42.22					
**86**	**G-OVX**	350.1	34.68	40.42	3.7912	2.3419	0.07247	0.5231	0.7519
**87**	**G-OVX**	353.4	34.49	40.67	6.8289	2.1743	0.0711	0.96	0.4823
**88**	**G-OVX**	373.8	34.19	40.36	7.6345	2.1019	0.06883	1.1091	0.44209
**89**	**G-OVX**	327.6	34.36	40					
**90**	**G-OVX**	428	35.51	42.07	6.7042	2.2158	0.0729	0.9193	0.5139
**91**	**G-OVX**	415.4	36.52	41.27	7.5474	2.1852	0.0681	1.1068	0.4924
**92**	**G-OVX**	403.4	35.98	39.4					
**93**	**G-OVX**	388.3	33.74	40.21	8.8345	2.2134	0.0767	1.1517	0.4349
**94**	**G-OVX**	384.5	35.17	40.32	11.2596	2.0434	0.0642	1.7516	0.32122
**95**	**G-OVX**	365.9	34.59	41.56					
	**AVG**	**386.1133**	**35.19333**	**40.65467**	**8.527256**	**2.1509**	**0.070767**	**1.2126**	**0.461269**
	**SD**	**30.91315**	**1.496733**	**1.075891**	**3.105943**	**0.126561**	**0.00359**	**0.469041**	**0.135467**
	**CV**	**8.006237**	**4.252888**	**2.646414**	**36.42371**	**5.884094**	**5.072887**	**38.68057**	**29.36837**
		0.627162	0.964018	0.706807	0.020447	0.087147	0.572448	0.027062	0.115107

DA
3.01747
2.76106
2.82291
3.07145
2.85945
2.96657
2.5839
2.58704
2.81693
2.64458
2.67554
2.75949
3.17439
**2.813934**
**0.204618**
**7.27159**
2.70318
2.69451
2.46441
3.18992
2.62261
2.78991
2.47177
**2.705187**
**0.245472**
**9.074111**
3.01697
2.81009
2.27167
2.78468
2.94271
2.54996
2.5433
2.83058
2.78407
3.23706
2.42158
2.45023
2.70554
**2.719111**
**0.26723**

**9.827831**
0.247933
1.937
2.03395
1.718
1.795
1.60825
1.819
1.82
1.61
1.836
**1.797467**
**0.139247**
**7.74685**
1.578
1.841
1.78
1.7938
1.8525
1.679
1.76
1.787
1.85
**1.769033**
**0.089749**
**5.073344**
0.613665
1.93847
1.852
2.0059
1.5624
1.88
2.0125
2.0888

2.134
**1.934259**
**0.178502**
**9.228448**
2.2255
1.987

1.5533
1.96458
1.7985
2.0508
1.7931
1.7841
1.8264
**1.887031**
**0.193551**
**10.25693**
0.608433

Animal		Crucible W	Dry Weigh	Dry Weigh	Ash Weigh	Ash Weigh	Ash %
**1**	**C-FR1**	10.5652	10.8567	0.2915	10.7355	0.1703	0.58422
**2**	**C-FR2**	9.9673	10.2657	0.2984	10.1428	0.1755	0.588137
**3**	**C-FR3**	10.1757	10.4952	0.3195	10.3543	0.1786	0.558998
**4**	**C-FR4**	10.1759	10.4831	0.3072	10.3472	0.1713	0.557617
**5**	**C-FR5**	10.0372	10.3122	0.275	10.1915	0.1543	0.561091
**6**	**C-FR6**	10.0613	10.3285	0.2672	10.2102	0.1489	0.55726
**7**	**C-FR7**	10.0301	10.3727	0.3426	10.2117	0.1816	0.530064
**8**	**C-FR8**	10.1023	10.5874	0.4851	10.3538	0.2515	0.51845
	**Average**	**10.13938**	**10.46269**	**0.323313**	**10.31838**	**0.179**	**0.55698**
	**SD**	**0.186487**	**0.192661**	**0.069628**	**0.187987**	**0.031456**	**0.023734**
	**CV**	**0.018392**	**0.018414**	**0.215359**	**0.018219**	**0.175734**	**0.042613**
	**Min**	**9.9673**	**10.2657**	**0.2672**	**10.1428**	**0.1489**	**0.51845**
	**Max**	**10.5652**	**10.8567**	**0.4851**	**10.7355**	**0.2515**	**0.588137**
**9**	**C**	10.3159	10.5911	0.2752	10.483	0.1671	0.607195
**10**	**C**	10.5641	10.8851	0.321	10.7519	0.1878	0.585047
**11**	**C**	9.9627	10.2748	0.3121	10.1206	0.1579	0.505928
**12**	**C**	10.3105	10.601	0.2905	10.4774	0.1669	0.574527
**13**	**C**	10.6566	10.9691	0.3125	10.8342	0.1776	0.56832
**14**	**C**	10.6606	10.8688	0.2082	10.7923	0.1317	0.632565
	**Average**	**10.41173**	**10.69832**	**0.286583**	**10.57657**	**0.164833**	**0.57893**
	**SD**	**0.270619**	**0.259849**	**0.041943**	**0.272085**	**0.019213**	**0.042835**
	**CV**	**0.025992**	**0.024289**	**0.146356**	**0.025725**	**0.116562**	**0.07399**
	**Min**	**9.9627**	**10.2748**	**0.2082**	**10.1206**	**0.1317**	**0.505928**
	**Max**	**10.6606**	**10.9691**	**0.321**	**10.8342**	**0.1878**	**0.632565**
**45**	**FR-G**	10.3415	10.6264	0.2849	10.5028	0.1613	0.566164
**46**	**FR-G**	10.0383	10.3091	0.2708	10.1864	0.1481	0.546898
**47**	**FR-G**	10.6591	10.9371	0.278	10.8035	0.1444	0.519424
**48**	**FR-G**	10.0321	10.2912	0.2591	10.1831	0.151	0.582787
**49**	**FR-G**	9.8041	10.0789	0.2748	9.9491	0.145	0.527656
**50**	**FR-G**	9.805	10.0919	0.2869	9.9652	0.1602	0.558383
**51**	**FR-G**	10.501	10.7419	0.2409	10.6386	0.1376	0.571191
**52**	**FR-G**	10.1518	10.422	0.2702	10.3015	0.1497	0.554034
	**Average**	**10.16661**	**10.43731**	**0.2707**	**10.31628**	**0.149663**	**0.553317**
	**SD**	**0.312113**	**0.308014**	**0.014914**	**0.309138**	**0.007979**	**0.021473**
	**CV**	**0.0307**	**0.029511**	**0.055095**	**0.029966**	**0.053313**	**0.038807**
	**Min**	**9.8041**	**10.0789**	**0.2409**	**9.9491**	**0.1376**	**0.519424**
	**Max**	**10.6591**	**10.9371**	**0.2869**	**10.8035**	**0.1613**	**0.582787**
**53**	**GNRH**	10.3161	10.6098	0.2937	10.4929	0.1768	0.601975
**54**	**GNRH**	10.0243	10.2618	0.2375	10.1714	0.1471	0.619368
**55**	**GNRH**	10.0614	10.3511	0.2897	10.2347	0.1733	0.598205
**56**	**GNRH**	9.9667	10.2681	0.3014	10.1479	0.1812	0.601194

**57**	**GNRH**	10.5008	10.79	0.2892	10.6742	0.1734	0.599585
**58**	**GNRH**	10.0371	10.3333	0.2962	10.2109	0.1738	0.586766
**59**	**GNRH**	10.1588	10.4326	0.2738	10.3267	0.1679	0.613221
**60**	**GNRH**	10.308	10.6032	0.2952	10.4828	0.1748	0.592141
**61**	**GNRH**	10.7745	11.1179	0.3434	10.9735	0.199	0.579499
**62**	**GNRH**	10.0368	10.3118	0.275	10.206	0.1692	0.615273
**63**	**GNRH**	9.8069	10.0934	0.2865	9.9792	0.1723	0.601396
**64**	**GNRH**	10.7757	11.0508	0.2751	10.9401	0.1644	0.597601
**65**	**GNRH**	10.1759	10.4368	0.2609	10.3352	0.1593	0.610579
**66**	**GNRH**	10.0597	10.3951	0.3354	10.249	0.1893	0.564401
	**Average**	**10.21448**	**10.50398**	**0.2895**	**10.38746**	**0.172986**	**0.598657**
	**SD**	**0.291988**	**0.300324**	**0.026953**	**0.295486**	**0.012406**	**0.014704**
	**CV**	**0.028586**	**0.028591**	**0.093101**	**0.028446**	**0.071718**	**0.024562**
	**Min**	**9.8069**	**10.0934**	**0.2375**	**9.9792**	**0.1471**	**0.564401**
	**Max**	**10.7757**	**11.1179**	**0.3434**	**10.9735**	**0.199**	**0.619368**
**15**	**C-R**						
**16**	**C-R**	10.0398	10.7607	0.7209	10.5322	0.4924	0.683035
**17**	**C-R**	10.178	10.996	0.818	10.7312	0.5532	0.676284
**18**	**C-R**	10.3481	11.0645	0.7164	10.8259	0.4778	0.666946
**19**	**C-R**						
**20**	**C-R**	10.4286	11.1298	0.7012	10.8943	0.4657	0.664147
**21**	**C-R**						
**22**	**C-R**	10.5037	11.1471	0.6434	10.9446	0.4409	0.685266
**23**	**C-R**	10.1785	10.9241	0.7456	10.6667	0.4882	0.654775
**24**	**C-R**	10.3482	11.055	0.7068	10.8233	0.4751	0.672184
**25**	**C-R**						
**26**	**C-R**						
**27**	**C-R**	10.4291	11.161	0.7319	10.9136	0.4845	0.661976
**28**	**C-R**	10.3477	10.9502	0.6025	10.7488	0.4011	0.665726
**29**	**C-R**						
	**Average**	**10.3113**	**11.02093**	**0.709633**	**10.78673**	**0.475433**	**0.670038**
	**SD**	**0.149085**	**0.12902**	**0.060878**	**0.131898**	**0.040925**	**0.010033**
	**CV**	**0.014458**	**0.011707**	**0.085788**	**0.012228**	**0.08608**	**0.014973**
	**Min**	**10.0398**	**10.7607**	**0.6025**	**10.5322**	**0.4011**	**0.654775**
	**Max**	**10.5037**	**11.161**	**0.818**	**10.9446**	**0.5532**	**0.685266**
**67**	**G-R**	9.776	10.4087	0.6327	10.1858	0.4098	0.6477
**68**	**G-R**	9.4921	10.1125	0.6204	9.9055	0.4134	0.666344
**69**	**G-R**	10.3177	11.0433	0.7256	10.7896	0.4719	0.650358
**70**	**G-R**	10.6625	11.3478	0.6853	11.1221	0.4596	0.670655
**71**	**G-R**	10.1611	10.9461	0.785	10.6815	0.5204	0.66293
**72**	**G-R**	9.8096	10.6217	0.8121	10.3546	0.545	0.6711
**73**	**G-R**						
**74**	**G-R**						

**75**	**G-R**	10.4312	10.5244	0.0932	10.489	0.0578	0.620172
**76**	**G-R**	10.3179	11.0263	0.7084	10.7922	0.4743	0.669537
**77**	**G-R**						
**78**	**G-R**	10.7769	11.5836	0.8067	11.318	0.5411	0.670757
**79**	**G-R**	10.034	10.7777	0.7437	10.5372	0.5032	0.676617
**80**	**G-R**	9.7753	10.5378	0.7625	10.2725	0.4972	0.652066
	**Average**	**10.1413**	**10.81181**	**0.670509**	**10.58618**	**0.444882**	**0.65984**
	**SD**	**0.404076**	**0.429779**	**0.201851**	**0.412696**	**0.136011**	**0.016314**
	**CV**	**0.039845**	**0.039751**	**0.301042**	**0.038984**	**0.305723**	**0.024725**
	**Min**	**9.4921**	**10.1125**	**0.0932**	**9.9055**	**0.0578**	**0.620172**
	**Max**	**10.7769**	**11.5836**	**0.8121**	**11.318**	**0.545**	**0.676617**

**30**	**C-OVX**	10.907	11.542	0.635	11.3292	0.4222	0.664882
**31**	**C-OVX**	9.2896	9.9419	0.6523	9.7102	0.4206	0.644795
**32**	**C-OVX**	10.3517	11.0486	0.6969	10.801	0.4493	0.644712
**33**	**C-OVX**	10.6646	11.3735	0.7089	11.1188	0.4542	0.640711
**34**	**C-OVX**	10.6672	11.3796	0.7124	11.1301	0.4629	0.649775
**35**	**C-OVX**	10.1641	10.771	0.6069	10.5684	0.4043	0.666172
**36**	**C-OVX**	10.778	11.4852	0.7072	11.2104	0.4324	0.611425
**37**	**C-OVX**	9.7777	10.5341	0.7564	10.2707	0.493	0.651772
**38**	**C-OVX**	10.3226	11.039	0.7164	10.7849	0.4623	0.64531
**39**	**C-OVX**	9.4952	10.1483	0.6531	9.9123	0.4171	0.638646
**40**	**C-OVX**	10.0361	10.8426	0.8065	10.5445	0.5084	0.630378
**41**	**C-OVX**	9.2878	9.9843	0.6965	9.7522	0.4644	0.666762
**42**	**C-OVX**	10.5068	11.1947	0.6879	10.9742	0.4674	0.679459
**43**	**C-OVX**	9.9744	10.6364	0.662	10.3987	0.4243	0.640937
**44**	**C-OVX**	10.5674	11.2765	0.7091	11.0256	0.4582	0.646171
	**Average**	**10.18601**	**10.87985**	**0.693833**	**10.63541**	**0.4494**	**0.648127**
	**SD**	**0.530993**	**0.535695**	**0.048967**	**0.532373**	**0.029211**	**0.016571**
	**CV**	**0.05213**	**0.049237**	**0.070575**	**0.050057**	**0.064999**	**0.025568**
	**Min**	**9.2878**	**9.9419**	**0.6069**	**9.7102**	**0.4043**	**0.611425**
	**Max**	**10.907**	**11.542**	**0.8065**	**11.3292**	**0.5084**	**0.679459**

**81**	**G-OVX**	9.7794	10.5434	0.764	10.2879	0.5085	0.665576
**82**	**G-OVX**						
**83**	**G-OVX**	9.289	9.9997	0.7107	9.7372	0.4482	0.630646
**84**	**G-OVX**						
**85**	**G-OVX**	10.5995	11.7373	1.1378	11.4872	0.8877	0.78019
**86**	**G-OVX**	9.4934	10.1446	0.6512	9.9012	0.4078	0.626229
**87**	**G-OVX**	10.3191	10.9986	0.6795	10.7554	0.4363	0.64209
**88**	**G-OVX**	10.3196	10.9966	0.677	10.9057	0.5861	
**89**	**G-OVX**	10.3495	11.1049	0.7554	10.8145	0.465	0.615568
**90**	**G-OVX**	10.0436	10.7893	0.7457	10.5289	0.4853	0.650798
**91**	**G-OVX**	9.9723	10.681	0.7087	10.4281	0.4558	0.643149
**92**	**G-OVX**	10.5053	11.2861	0.7808	11.0057	0.5004	0.640881

**93**	**G-OVX**	10.0418	10.7113	0.6695	10.472	0.4302	0.642569
**94**	**G-OVX**	10.0656	10.749	0.6834	10.487	0.4214	0.616623
**95**	**G-OVX**	10.3209	11.0638	0.7429	10.7617	0.4408	
	**Average**	**10.08454**	**10.8312**	**0.746662**	**10.5825**	**0.497962**	**0.650393**
	**SD**	**0.384108**	**0.456749**	**0.124403**	**0.459293**	**0.126139**	**0.045473**
	**CV**	**0.038089**	**0.04217**	**0.166612**	**0.043401**	**0.253312**	**0.069917**
	**Min**	**9.289**	**9.9997**	**0.6512**	**9.7372**	**0.4078**	**0.615568**
	**Max**	**10.5995**	**11.7373**	**1.1378**	**11.4872**	**0.8877**	**0.78019**
